# Optimal Level of Supplemental Manganese for Yellow-Feathered Broilers during the Growth Phase

**DOI:** 10.3390/ani11051389

**Published:** 2021-05-13

**Authors:** Yibing Wang, Zhongyong Gou, Xiajing Lin, Qiuli Fan, Jinling Ye, Shouqun Jiang

**Affiliations:** State Key Laboratory of Livestock and Poultry Breeding, Key Laboratory of Animal Nutrition and Feed Science in South China, Ministry of Agriculture and Rural Affairs, Guangdong Provincial Key Laboratory of Animal Breeding and Nutrition, Institute of Animal Science, Guangdong Academy of Agricultural Sciences, Guangzhou 510640, China; wangyibing77@163.com (Y.W.); Gouzhongyong@gdaas.cn (Z.G.); Linxiajing@gdaas.cn (X.L.); fanqiuli_829@163.com (Q.F.); YeJL2014@163.com (J.Y.)

**Keywords:** growth performance, manganese, meat quality, yellow-feathered broiler, tibial development

## Abstract

**Simple Summary:**

Manganese is an indispensable trace element, necessary for the normal development and activity of tissues such as bones. The low Mn content in corn–soybean meal diets used in production and the inefficient intestinal absorption of Mn in broilers calls for the need for optimizing the supplemental provision of Mn to broilers. The current study examined the effect of an optimized dietary supplemental Mn for growth performance, tibial characteristics, immune function and meat quality of yellow-feathered broilers and recommended that optimal supplementation with Mn in diets for birds to achieve the best performance was 52 (d 1 to d 21), 60 (d 22 to d 42), and 68 mg/kg (d 43 to d 63). This study provides a rational recommendation for the appropriate dietary nutrient levels and provides a scientific basis for establishing nutritional standards of yellow-feathered broilers.

**Abstract:**

This experiment investigated the effect of an optimized supplemental dietary manganese (Mn) on growth performance, tibial characteristics, immune function and meat quality, of yellow-feathered broilers. In three rearing periods, birds were fed for 21-d periods, from d 1 (starter), d 22 (grower) and d 43 (finisher), respectively, with basal diets (containing 16, 17, and 14 mg/kg analyzed Mn, respectively) supplemented with 0, 20, 40, 60, 80, 100, 120 and 140 mg/kg Mn. For starter phase broilers, supplemental manganese affected feed to gain ratio (**F/G**), and the minimum value was observed with 120 mg/kg manganese. During the grower phase, ADG increased quadratically (*p* < 0.05) with supplemental Mn and was maximal with 54 mg/kg additional manganese estimated using the regression equation. There was no influence of supplemental manganese on growth performance of broilers during the finisher phase (*p* > 0.05). The thymic relative weight of broilers were linearly (*p* < 0.05) and quadratically (*p* < 0.05) increased with supplemental Mn and maxima were obtained with 95 and 110 mg/kg additional Mn at 42 d and 63 d. The bone density of the tibia in broilers at d 21, 42 and 63 were increased quadratically (*p* < 0.05) by supplemental Mn, and optimal supplementation for the three phases was 52, 60 and 68 mg/kg, respectively. The weight, diameter, breaking strength and bone density of the tibia of 63-d broilers were influenced (*p* < 0.05) by supplemental manganese. The lightness (L*) value (linear, *p* < 0.05) and yellowness (b*) value (*p* < 0.05) of the breast muscle were decreased by dietary manganese supplementation, and the optimal supplementation, based on L*, was 86 mg/kg. In conclusion, supplemental Mn affected the growth performance, thymic relative weight, tibial characteristics, and the meat color of yellow-feathered broilers. From the quadratic regressions, the optimal supplementation of yellow-feathered broilers at the starter, grower and finisher phases to achieve the best performance was 52, 60, and 68 mg/kg, respectively.

## 1. Introduction

Manganese (Mn) is an indispensable trace element, necessary for the normal development and activity of tissues [1]. It is an important component of enzymes involved in growth, carbohydrate and lipid metabolism, and blood clotting [2,3]. Earlier study has shown that dietary Mn is essential for the prevention of the deformity of the tibiae and metatarsi of chickens [4]. Numerous recent studies have proved that Mn plays an important role in immune function [5], meat quality [6] and reproductive performance of broilers [2]. Due to the low Mn content in corn–soybean meal diets [7] used in production and the inefficient intestinal absorption of Mn in broilers [8], it is necessary to supplement diets with Mn [2]. It is also known that exposure to excessive Mn is related to severe damage to the liver, lungs and the reproductive and immune systems in broilers [9]. All the foregoing indicates the need for optimizing supplemental provision of Mn to broilers.

As the very important strain of broilers, the yellow-feathered broiler is famous for the great meat quality. Nowadays, the production of yellow-feathered broilers has been approximately 4 billion annually, almost same as white-feathered broilers. Work from this group optimized supplemental Mn for yellow-feathered breeder hens during the peak period of laying at 90 to 135 mg/kg [10], while the nutritional requirements of yellow-feathered broilers during growth to market size remains incomplete. In the current study, a hypothesis was formulated that dietary supplemental Mn affected the growth performance, tibial development, immune function and meat quality of yellow-feathered broilers in dose-dependence, and diets with different supplementations of Mn were used to determine the optimized dietary supplemental Mn for yellow-feathered broilers. The results will provide a rational recommendation for the appropriate dietary nutrient levels for yellow-feathered broilers.

## 2. Material and Methods

### 2.1. Experimental Design

Chinese yellow-feathered male broilers (Lingnan, rapidly growing yellow-feathered broilers) were used to assess effects of supplemental level of Mn during starter, grower and finisher phases of growth.

Starter phase (d 1 to 21): 1920 hatchlings were randomly divided into 8 groups, 6 replicates per treatment, 40 birds per replicate, and birds were fed a basal diet (16 mg/kg Mn) supplemented with 0, 20, 40, 60, 80, 100, 120 or 140 mg/kg Mn (MnSO_4_).

Grower phase (d 22 to 42): 1,440 broilers were raised during the starter phase on a diet containing 20 mg/kg Mn (to reduce Mn deposition in organs) then allocated to the same 8 supplemental treatments, each with 6 replicates of 30 birds; the basal grower diet contained 17 mg/kg Mn. Broilers were supplemented with the same levels of Mn as above.

Finisher phase (d 43 to 63): Broilers that had previously received diets containing 80 mg/kg Mn at the starter phase (the current recommendation for starter phase) and 20 mg/kg Mn at the grower phase (to reduce Mn deposition in organs) were used. Birds at 43 d (*n* = 800) were randomly assigned to the 8 supplemental Mn treatments as before, using a basal diet containing 14 mg/kg Mn; each treatment consisted of 5 replicates of 20 broilers.

### 2.2. Experimental Diets and Chicken Husbandry

The diets were formulated as Chinese Feeding Standard of Chicken recommended [11], with the exception of Mn. Details of ingredient composition and calculated nutrient contents of basal diets were given (Table 1). The Mn concentration in basal diets was determined as described previously [12] and is showed in Table 2. In brief, diets were weighed and digested with HNO_3_ and HClO_4_, and Mn concentration was determined by inductively coupled argon plasma spectroscopy [12].

Water and diets were provided ad libitum throughout. The room temperature was kept at 32 to 34 °C at the first 3 days and reduced to a final temperature of 26 °C (2 °C per week). The light cycle was 24L:0D from d 1 to d 2, 23L:1D from d 3 to d 10, and 18L:6D from d 11 with incandescent bulbs. Birds were raised in floor pens with wood shavings litter, and the stocking density was 0.20, 0.27, and 0.40 m^2^/bird during the three phases of growth, respectively.

### 2.3. Measurement of Growth Performance and Carcass Traits

Birds were weighed at the beginning and end of each 3-wk growth phase on a per replicate basis. The final body weight, average daily gain, average daily feed intake and feed/gain ratio were calculated as previously described [13].

At the end of each phase, 2 birds close to average BW per replicate were deprived of feed overnight and weighed immediately prior to slaughter. The birds were electrically stunned and exsanguinated. The spleen, thymus and bursa of Fabricius were dissected, blotted and weighed. The relative weight of immune organs was calculated. Relative weight = the immune organ weight/live weight × 100%.

### 2.4. Measurement of Tibial Charcteristics

Two pairs of tibias of the birds dissected above were collected for analyses. Tibias were cleaned from all adherent tissues. For the left tibia, the bone breaking strength was determined with a materials tester (Instron 4411, Instron Corporation, Grove City, PA, USA), as described by Wang et al. [10]. For the right tibia, it was blotted dry with paper towels and then weighted; the length and diameter were measured with a caliper; the mineral density was measured with an X-ray osteodensitometer (Lunar Prodigy, General Electric Company, Fairfield, CT). Tibias (2 g) were ashed at 600 °C to constant weight (less than 0.5 mg before and after incineration) [14], and the content of Mn in bone ash was measured using inductively coupled argon plasma spectroscopy, according to the method described previously [12].

### 2.5. Determination of Meat Quality

Breast muscles (the whole left *pectoralis major*) of the chosen birds were collected and kept at 4 °C. Shear force 45 min post-mortem, and drip loss 24 h post-mortem was determined as previously described [13]. In brief, muscle samples were cut, weighed and placed in a plastic bag filled with air in 4 °C for 24 h. The drip loss was determined: drip loss = (weight_24h_ − weight_0h_)/weight_24h_ * 100% [13]. The muscles were cooked to an internal temperature of 70 °C. After cooling to room temperature, segments 1 cm^2^ were cut perpendicular to the fiber orientation of the muscle then 10 sections about 3 cm thick were cut parallel to the fiber orientation to determine the shear force [13].

### 2.6. Statistical Analysis

A replicate (pen for the determination of growth performance and bird for other indicators) served as the experimental unit. The effects of Mn supplementation were analyzed by a one-way ANOVA procedure (SPSS Inc., Chicago, IL, USA). Means were separated by Duncan’s multiple range test. Where appropriate, polynomial regressions were fitted to test for linear and quadratic effects in response to Mn supplementation [15]. When a significant quadratic component was demonstrated (*p* < 0.05), regression analyses were used to estimate supplemental Mn optimization (the maximum response from a quadratic model).

## 3. Results

### 3.1. Growth Performance

As present in Table 3, for starter phase broilers (1 to 21 d) there was a significant effect of Mn supplementation on the feed to gain ratio (F/G) (*p* < 0.05), with 120 mg/kg additional Mn decreasing F/G (*p* < 0.05). Manganese supplementation did not affect body weight (BW), average daily gain (ADG) or average daily feed intake (ADFI) (*p* > 0.05) during the starter phase. During the grower phase (d 22 to d 42), ADG was increased quadratically (*p* < 0.05) by supplemental Mn. There were tendencies for BW (*p* = 0.071) and F/G (*p* = 0.064) to be affected. There were no significant effects of supplemental Mn on the growth performance of broilers at 43 to 63 d (*p* > 0.05).

### 3.2. Immune Organ Relative Weight

For 21 d broilers (Table 4), dietary Mn supplementation did not affect relative weight of immune organs (*p* > 0.05). There were both linear (*p* < 0.05) and quadratic (*p* < 0.05) effects of supplemental Mn on relative weights of the thymus at both 42 d and 63 d. In detail, supplementation with 20, 80, 100 or 120 mg/kg Mn increased the relative weight of the thymus of broilers at 42 d, and supplementation with 60, 100, 120 or 140 mg/kg Mn increased the relative weight of the thymus of broilers at 63 d.

### 3.3. Tibial Characteristics

As given in Table 5, for the starter phase there were both linear (*p* < 0.05) and quadratic (*p* < 0.05) effects of supplemental Mn on the bone density of the tibia. During the grower phase, the diameter and bone density of the tibia were increased (*p* < 0.05) by supplementation of Mn. Supplementation with 60 mg/kg Mn increased the diameter of the tibia compared with broilers in other treatments (*p* < 0.05). Bone density was quadratically increased (*p* < 0.05) with increasing Mn. Compared with birds in the control group, bone density was increased (*p* < 0.05) when birds were supplemented with 20, 40 or 100 mg/kg Mn. For finisher phase broilers, the weight, diameter, breaking strength and bone density of the tibia were affected (*p* < 0.05) by supplemental Mn. Supplementing with 100 mg/kg Mn significantly increased the weight, diameter and breaking strength compared with other treatments (*p* < 0.05). Bone density was quadratically increased (*p* < 0.05) with increasing Mn. Compared with birds in the control group, bone density was increased (*p* < 0.05) when birds were supplemented with 80 or 100 mg/kg Mn.

### 3.4. Manganese Deposition in Tibia

The effects of dietary supplemental Mn on Mn deposition in the tibia are presented in Table 6. There were both linear (*p* < 0.001) and quadratic (*p* < 0.001) effects of supplemental Mn on Mn content in the tibia of broilers in all three phases, where the highest values were observed with 120 mg/kg additional Mn in the starter phase, 100, 120 and 140 additional Mn in the grower phase and 140 mg/kg additional Mn in the finisher phase, respectively.

### 3.5. Meat Quality

The effects of dietary supplemental Mn on the meat quality of 63-d broilers are presented in Table 7. Dietary supplemental Mn had no significant influence on the shear force and drip loss of breast muscle (*p* > 0.05).

### 3.6. Estimations of the Optimal Level of Supplemental Mn

The optimal levels of supplemental Mn of yellow-feathered broilers from the quadratic regressions (the maximum response from a quadratic model) are shown in Table 8. The optimal levels of supplemental Mn estimated using the regression equation were 26 mg/kg for F/G, 52 mg/kg for bone density, and 198 mg/kg for Mn content in the tibia for broilers aged 1 to 21 d. During the grower phase, the optimal supplementation was 54 mg/kg for ADG, 95 mg/kg for the thymic index, 60 mg/kg for bone density, and 162 mg/kg for Mn content in the tibia. During the finisher phase, optima were 110 mg/kg for the thymic index, and 68 mg/kg for bone density.

## 4. Discussion

Manganese takes a crucial part in biological processes, including the metabolism of lipid, protein, and carbohydrate [16]. Several studies showed that Mn improved the growth performance of broilers. Ross 708 male broilers fed corn–soy diets with elevated levels of Mn at 80, 120 or 160 mg/kg had improved feed conversion ratio [17]. Manganese at 45 to 130 mg/kg significantly increased the BW of broilers from 1 to 49 d [7]. For the Gushi Broiler, another Chinese yellow-feathered strain, the highest weight gain was obtained when chicks received 90 mg/kg dietary Mn [18]. The effects of supplemental Mn on the performance of broilers were inconsistent, however, and several studies failed to demonstrate any beneficial effects of supplemented Mn on BW or F/G [19,20]. The current study with yellow-feathered broilers showed that supplemental Mn improved the growth performance during the starter and grower phase broilers but was without effect on that during the finisher phase. Considering growth performance, supplementation with 120 and 54 mg/kg was optimal for yellow-feathered broilers at the starter and grower phases, achieving the lowest F/G or highest ADG, respectively.

Manganese has been proved to be important in supporting normal immune functions in broilers [21]. The present study indicated that there were benefits of supplemental Mn on thymic relative weights at both 42 d and 63 d. A previous study showed that the supplementation of 75 to 100 mg/kg Mn (to basal diets containing 23.3 to 26.4 mg/kg Mn) of chickens enhanced the humoral immune response and increased antibody titers against Newcastle disease virus [19], and for broilers, Mn supplementation also enhanced the antibody titer to sheep red blood cells and improved the cell immunity of basophil sensitization to plant lectins [5,16]. The reason for Mn improving immunity may be from its contribution to activity of Mn superoxide dismutase (MnSOD) [22], which is vital for the integrity of macrophages [19], as MnSOD interacts with heterophils and macrophages through plasma membrane cells which act in the immune response [21]. This effect of Mn in yellow-feathered broilers needs further study because supplemental Mn in mice enhanced phagocytosis of macrophages and natural killer cells by increasing IFN-γ [16] and increased gene expression of *IFN-*γ, *IL-1β*, *IL-6*, and *IL-8* in rat microglia and human monocyte-derived macrophages [16,23]. On the other hand, excessive Mn accumulated in the immune organs of birds exposed to high Mn and disturbed the balance of the microelements and induced immune suppression at the molecular level [9]. Exposure to Mn particles in vitro was suggested to adversely affect the adaptive cellular response in viral-induced IFN-γ production [24]. In the current research, optimal levels of supplemental Mn for the thymic index of yellow-feathered broilers were 94 and 110 mg/kg during the grower and finisher phases, achieving the most relative weight of the thymus.

Mn is essential for normal bone development in young chicks. Manganese is absorbed from the intestinal lumen [25] into the hepatic portal vein and the bulk of the Mn accumulates in bone [26]. Receiving 30 to 120 mg/kg dietary Mn induced an increase in tibial Mn content of cockerel chicks [20], and the plasma [27], hepatic, renal, and tibial [17,28] contents were higher in Mn-supplemented broilers than those in controls. Similarly, Mn content in the tibia of yellow-feathered broilers in all three phases examined here were obviously responsive to increasing levels of supplemental Mn. It is worth noting that the optimal levels of Mn to obtain the highest Mn content in tibia are 198 and 162 mg/kg for broilers during the starter and grower phases; however, in that situation, the dietary Mn level was considered to be too high so as to have negative influence on growth or health of broilers. Therefore, Mn content in the tibia is not suitable to be used as a sensitive indicator to evaluate the optimal level of supplemental Mn.

Dietary Mn is known to have profound effects on the skeleton. Manganese insufficiencies resulted in malformation of the epiphyseal plate of the tibia [29], an enlargement of the intertarsal joint, and either twisting or shortening of the tibia [4,8]; furthermore, Mn deficiency is also related to osteoporosis [8], and Mn-deficient diets lead to decreased ash content and length of leg bones in chicks [30]. It was found here that dietary supplementation with Mn improved tibial parameters during all the growth phases. Similarly, broilers fed Mn at 160 mg/kg exhibited improved tibial breaking strength [17]. It has been demonstrated that manganese is involved in bone regulation through many paths. Mangnese is important in the synthesis of mucopolysaccharides [2,4,5,26] which are major constituents of bone extracellular matrixes and central to the development of the physical problems [30]. Manganese also participates through its contribution to enzyme activity within metabolic pathways involved in the formation of the skeletal system [31]. Oliveira et al. [31] suggested that improvements in the concentrations of Mn used as components of metalloenzymes are necessary for the synthesis of connective tissue. In addition, Mn played a significant role in the vitality of osteoblasts by regulating relative mRNA expression levels of *RANKL* (receptor activator of nuclear factor κB ligand) and *OPG* (osteoprotegerin), thus affected the normal development of the tibia [8]. It is worth noting that excessive Mn may impair the absorption of other minerals and is related to severe damage to the physiological process of broilers [7,9]. In the current research, the bone density of broilers in the starter phase significantly decreased when supplemented with 140 mg/kg, indicating that 140 mg/kg might be excessive for broilers in the starter phase. The present study indicated that 52, 60, and 68 mg/kg supplemental Mn met the requirements of yellow-feathered broilers for bone density at the three growth phases.

In the current study, supplemental Mn did not affect the drip loss and shear force of breast muscle, which was similar with the research of Lu et al. [32]. For color attributes, the L* and b* value of breast muscle were influenced by dietary Mn supplementation. Yang et al. [3] suggested optimal dietary Mn supplementation was important for improving the muscle quality variables of chickens and 40 mg/kg additional Mn decreased L* value in breast muscle. Lu et al. [6] also showed that Mn decreased b* value of leg muscle and influenced pH in breast muscle of Arbor Acres male broilers. An important factor contributing to reduced meat quality is lipid oxidation [32]. Manganese is a component of MnSOD, the primary antioxidant enzyme protecting cells from oxidative stress, enhancing the antioxidant ability of scavenging excessive reactive oxygen species (ROS) and reducing lipid peroxidation in broilers [33]. It was suggested that supplemental Mn might improve the meat quality of the yellow-feathered broilers studied here, probably by affecting MnSOD activities and reducing the content of malondialdehyde, an indicator reflecting the extent of lipid oxidation in meat, as noted by Lu et al. [32] and Zhang et al. [34]. For further study of Mn on the meat quality of yellow-feathered broilers, pH value and indicators for color attributes in muscles of birds post mortem should be considered.

## 5. Conclusions

Dietary Mn supplementation affected growth performance, thymus relative weight, tibial characteristics and color attributes of the meat of yellow-feathered broilers. Considering these markers together, from the quadratic regressions, optimal supplementation with Mn in diets for birds to achieve the best performance was 52 (d 1 to d 21, 16 mg/kg in basal diet), 60 (d 22 to d 42, 17 mg/kg in basal diet), and 68 mg/kg (d 43 to d 63, 14 mg/kg in basal diet).

## Figures and Tables

**Table 1:** Composition and nutrient levels of the basal diets.

	1 to 21 d	22 to 42 d	43 to 63 d
Ingredients, %			
Corn	61.50	66.70	74.82
Soybean meal	31.30	25.60	16.60
Corn gluten meal	1.00	1.21	2.00
Soybean oil	1.45	2.10	2.40
Limestone	1.90	1.73	1.61
CaHPO_4_	1.28	1.10	0.93
NaCl	0.30	0.30	0.30
*DL*-Methionine	0.17	0.11	0.08
*L*-Lysine·HCl (78%)	0.10	0.15	0.26
Vitamin and mineral Premix ^1^	1.00	1.00	1.00
Total	100.00	100.00	100.00
Nutrient Levels ^2^			
ME (kcal/kg)	2900	3000	3100
CP ^3^	21.00	19.00	16.00
CP (Analysed)	21.34	19.15	15.92
Ca	1.00	0.90	0.80
Ca (Analysed)	1.03	0.88	0.80
Available P	0.45	0.40	0.35
Ca/Available P	2.29	2.20	2.29
Lys	1.05	0.98	0.85
Met	0.50	0.40	0.34
Met+Cys	0.85	0.72	0.65
Mn, mg/kg	17	17	14
Mn, mg/kg (Analysed)	16	17	14

^1^ Premix for three phases was formulated according to the nutritional levels (except Mn) previously described [13], moreover, premix provided 15,000 IU/kg (the starter and grower phases) and 10,000 IU/kg (the finisher phase) vitamin A of diets. ^2^ Nutrient levels were calculated values. ^3^ CP = crude protein.

**Table 2:** Analyzed manganese (Mn) contents in experimental diets.

Added Mn, mg/kg	Analyzed Mn Contents, mg/kg		
	1 to 21 d	22 to 42 d	43 to 63 d
0	16	17	14
20	32	37	31
40	55	53	52
60	72	75	76
80	96	97	95
100	117	115	116
120	133	138	136
140	156	153	154

**Table 3:** Effects of dietary supplemental manganese on performance of yellow-feathered broilers.

Days of Age	Variable	Manganese Supplemental Level, mg/kg								SEM	*p* Value ^1^		
		0	20	40	60	80	100	120	140		Mn	Linear	Quadratic
1 to 21	Initial body weight/g	37.5	37.5	37.5	37.5	37.5	37.5	37.5	37.5	0.3	-		
	Final body weight/g	451.0	471.5	454.5	452.3	445.6	442.9	463.2	458.8	3.8	0.133		
	Average daily gain/g	21.87	22.45	21.90	21.76	21.38	21.27	22.06	21.85	0.11	0.160		
	Average daily feed intake/g	36.73	37.35	36.85	37.04	36.20	35.81	36.18	36.13	0.16	0.238		
	Feed/gain	1.69 ^ab^	1.69 ^ab^	1.70 ^a^	1.70 ^a^	1.69 ^ab^	1.66 ^bc^	1.64 ^c^	1.66 ^bc^	0.01	0.021	0.001	0.102
22 to 42	Initial body weight/g	471.7	471.7	471.7	471.7	471.7	471.7	471.7	471.7	1.2	-		
	Final body weight/g	1224.2	1249.1	1222.7	1238.3	1230.8	1238.6	1227.8	1191.9	12.8	0.071		
	Average daily gain/g	35.78 ^ab^	37.03 ^a^	35.78 ^ab^	36.52 ^a^	36.16 ^a^	36.54 ^a^	36.02 ^a^	34.31 ^b^	0.21	0.047	0.063	0.016
	Average daily feed intake/g	86.60	89.03	85.65	85.04	85.57	87.45	84.39	87.65	0.51	0.310		
	Feed/ gain	2.41 ^b^	2.41 ^b^	2.40 ^b^	2.33 ^b^	2.37 ^b^	2.40 ^b^	2.34 ^b^	2.56 ^a^	0.08	0.064		
43 to 63	Initial body weight/g	1246.8	1246.8	1246.8	1246.8	1246.8	1246.8	1246.8	1246.8	4.5	-		
	Final body weight/g	2530.9	2522.5	2507.3	2510.4	2505.3	2501.6	2474.4	2478.8	20.7	0.550		
	Average daily gain/g	60.59	61.31	60.02	60.17	59.93	59.75	58.46	58.67	2.20	0.527		
	Average daily feed intake/g	159.80	158.40	161.20	164.00	159.00	165.20	158.00	159.80	5.89	0.451		
	Feed/gain	2.61	2.61	2.69	2.73	2.65	2.77	2.71	2.73	0.12	0.294		

^a–c^ Within a row, means with different lowercase superscripts differ significantly (*p* < 0.05). ^1^ Linear and quadratic effects were tested only when manganese levels were significant.

**Table 4:** Effects of dietary supplemental manganese on relative weight of immune organs of yellow-feathered broilers.

Days of Age	Relative Weight, % of BW	Manganese Supplemental Level, mg/kg								SEM	*p* Value ^1^		
		0	20	40	60	80	100	120	140		Mn	Linear	Quadratic
21	Thymus	5.90	6.11	4.63	4.57	5.50	5.47	5.14	4.99	0.63	0.140		
	Spleen	1.35	1.23	1.20	1.36	1.24	1.05	1.24	1.25	0.04	0.702		
	Bursa of Fabricius	1.39	1.55	1.13	1.49	1.46	1.52	1.23	1.81	0.12	0.314		
42	Thymus	2.47 ^c^	3.43 ^ab^	2.53 ^c^	2.98 ^bc^	3.64 ^ab^	4.01 ^a^	3.68 ^ab^	2.89 ^bc^	0.95	0.002	0.030	0.019
	Spleen	2.30	2.10	2.22	2.15	2.42	2.38	2.53	2.38	0.51	0.536		
	Bursa of Fabricius	0.92	1.04	1.18	0.98	1.20	1.26	1.14	1.13	0.32	0.364		
63	Thymus	0.22 ^b^	0.31 ^ab^	0.30 ^ab^	0.33 ^a^	0.29 ^ab^	0.40 ^a^	0.35 ^a^	0.34 ^a^	0.10	0.038	0.007	0.009
	Spleen	0.15	0.14	0.15	0.15	0.15	0.13	0.14	0.16	0.03	0.711		
	Bursa of Fabricius	0.14	0.13	0.12	0.16	0.14	0.14	0.15	0.14	0.04	0.608		

^a–c^ Within a row, means with different lowercase superscripts differ significantly (*p* < 0.05) ^1^ Linear and quadratic effects were tested only when manganese levels were significant.

**Table 5:** Effects of dietary supplemental manganese on tibial characteristics of yellow-feathered broilers.

Days of Age	Variable	Manganese Supplemental Level, mg/kg								SEM	*p* Value ^1^		
		0	20	40	60	80	100	120	140		Mn	Linear	Quadratic
21	Weight, g	5.21	5.42	5.23	5.24	5.16	5.18	5.37	5.33	0.05	0.301		
	Length, mm	72.74	72.89	71.58	71.88	71.26	72.48	72.97	73.08	1.41	0.331		
	Diameter, mm	4.40	4.43	4.40	4.35	4.31	4.31	4.48	4.40	0.01	0.052		
	Breaking strength, kgf	12.31	11.56	11.55	12.51	11.85	10.22	11.56	11.10	1.04	0.121		
	Bone density, g/cm^2^	0.112 ^ab^	0.111 ^ab^	0.113 ^ab^	0.119 ^a^	0.116 ^a^	0.110 ^ab^	0.111 ^ab^	0.105 ^b^	0.01	0.018	0.022	0.004
42	Weight, g	12.72	12.68	12.67	12.80	12.63	12.47	12.28	12.60	0.45	0.180		
	Length, mm	103.02	103.76	103.02	103.29	102.76	103.61	103.77	102.74	1.73	0.848		
	Diameter, mm	6.21 ^c^	6.27 ^bc^	6.25 ^bc^	6.81 ^a^	6.45 ^b^	6.29 ^bc^	6.43 ^b^	6.44 ^b^	0.15	<0.001	0.236	0.157
	Breaking strength, kgf	20.41	20.19	22.89	19.98	19.71	21.01	20.28	18.89	2.64	0.103		
	Bone density, g/cm^2^	0.132 ^b^	0.140 ^a^	0.141 ^a^	0.134 ^ab^	0.137 ^ab^	0.141 ^a^	0.135 ^ab^	0.128 ^b^	0.01	0.044	0.209	0.028
63	Weight, g	25.90 ^b^	24.95 ^b^	25.00 ^b^	25.77 ^b^	25.71 ^b^	28.08 ^a^	25.30 ^b^	26.02 ^b^	1.56	0.001	0.101	0.238
	Length, mm	135.80	135.63	136.84	137.41	135.54	137.52	136.55	135.58	2.13	0.229		
	Diameter, mm	9.95 ^b^	9.48 ^b^	9.50 ^b^	9.89 ^b^	9.86 ^b^	11.04 ^a^	9.66 ^b^	10.01 ^b^	0.25	0.001	0.101	0.238
	Breaking strength, kgf	31.85 ^c^	30.41 ^c^	28.86 ^c^	28.50 ^c^	35.23 ^b^	44.14 ^a^	28.46 ^c^	31.10 ^c^	6.96	<0.001	0.394	0.441
	Bone density, g/cm^2^	0.143 ^b^	0.159 ^ab^	0.163 ^ab^	0.160 ^ab^	0.169 ^a^	0.165 ^a^	0.146 ^b^	0.152 ^b^	0.01	<0.001	0.742	0.046

^a–c^ Within a row, means with different lowercase superscripts differ significantly (*p* < 0.05) ^1^ Linear and quadratic effects were tested only when manganese levels were significant.

**Table 6:** Effects of dietary supplemental manganese on manganese content in tibia of yellow-feathered broilers(μg/g). ^1.^

Days of Age	Manganese Supplemental Level, mg/kg								SEM	*p* Value ^1^		
	0	20	40	60	80	100	120	140		Mn	Linear	Quadratic
21	2.62 ^e^	2.88 ^de^	3.04 ^d^	3.76 ^c^	4.14 ^b^	3.91 ^bc^	4.81 ^a^	4.35 ^b^	0.79	<0.001	<0.001	<0.001
42	2.80 ^e^	3.27 ^d^	3.79 ^c^	3.81 ^c^	4.28 ^b^	4.45 ^ab^	4.56 ^ab^	4.71 ^a^	0.75	<0.001	<0.001	<0.001
63	2.72 ^d^	2.85 ^cd^	3.10 ^c^	3.33 ^bc^	3.62 ^b^	3.48 ^b^	3.61^b^	4.02 ^a^	0.52	<0.001	<0.001	<0.001

^a–^^e^ Within a row, means with different lowercase superscripts differ significantly (*p* < 0.05) ^1^ Linear and quadratic effects were tested only when manganese levels were significant.

**Table 7:** Effects of dietary supplemental manganese on meat quality of 63-d yellow-feathered broilers.

Variable	Manganese Supplemental Level, mg/kg								SEM	*p* Value ^1^		
	0	20	40	60	80	100	120	140		Mn	Linear	Quadratic
Shear force, kgf	3.88	3.70	3.19	3.39	4.00	3.59	3.35	3.09	0.72	0.053		
Drip loss, %	3.16	3.31	3.56	3.51	3.13	3.40	3.17	3.03	0.52	0.166		

^1^ Linear and quadratic effects were tested only when manganese levels were significant.

**Table 8:** Estimations of the optimal level of supplemental manganese (Mn) based on quadratic regressions ^1.^

Days of Age	Variables	Regression Equation ^1^	R^2^	*p* Value	Mn Requirement ^2^, mg/kg
1 to 21 d	Bone density, g/cm^2^	Y = 0.112 − 0.00000128X^2^ + 0.000133X	0.163	0.004	52
	Manganese content in tibia, g/g	Y = 2.49 − 0.0000576X^2^ + 0.0228X	0.750	<0.001	198
22 to 42 d	Average daily gain, g	Y = 35.90 − 0.000245X^2^ + 0.0263X	0.172	0.026	54
	Thymic relative weight, %	Y = 2.47 − 0.000113X^2^ + 0.0214X	0.123	0.019	95
	Bone density, g/cm^2^	Y = 0.133 − 0.00000160X^2^ + 0.000193X	0.198	0.023	60
	Manganese content in tibia, μg/g	Y = 2.83 − 0.0000716X^2^ + 0.0233X	0.686	<0.001	162
43 to 63 d	Thymic relative weight, %	Y = 0.238 − 0.00000986X^2^ + 0.00217X	0.151	0.009	110
	Bone density, g/cm^2^	Y = 0.151 − 0.00000235X^2^ + 0.000320X	0.294	0.046	68

^1^ Y is the dependent variable and X are the dietary manganese supplemental levels (mg/kg). ^2^ Dietary manganese requirement = the optimal supplemental manganese concentration according to each regression equation (mg/kg).

## Data Availability

Not applicable.

## References

[1] Wang J., Wang Z.Y., Wang Z.J., Liu R., Liu S.Q., Wang L. (2015). Effects of manganese deficiency on chondrocyte development in tibia growth plate of arbor acres chicks. J. Bone Miner. Metab..

[2] Xie J., Tian C., Zhu Y., Zhang L., Lu L., Luo X. (2014). Effects of inorganic and organic manganese supplementation on gonadotropin-releasing hormone-l and follicle-stimulating hormone expression and reproductive performance of broiler breeder hens. Poult. Sci..

[3] Yang X.J., Sun X.X., Li C.Y., Wu X.H., Yao J.H. (2011). Effects of copper, iron, zinc, and manganese supplementation in a corn and soybean meal diet on the growth performance, meat quality, and immune responses of broiler chickens. J. Appl. Poult. Res..

[4] Wilgus H.S., Norris L.C., Heusee G.F. (1937). The role of manganese and certain other trace elements in the prevention of perosis. J. Gynécol. Obst. Biol. Reprod..

[5] Gajula S.S., Chelasani V.K., Panda A.K., Mantena V.L.N.R., Savaram R.R. (2011). Effect of supplemental inorganic Zn and Mn and their interactions on the performance of broiler chicken, mineral bioavailability, and immune response. Biol. Trace Elem. Res..

[6] Lu L., Ji C., Luo X.G., Liu B., Yu S.X. (2006). The effect of supplemental manganese in broiler diets on abdominal fat deposition and meat quality. Anim. Feed Sci. Technol..

[7] Ta N. (2005). Effects of the Different Sources and Levels of Diet Manganese on Growth Performance and Immune Functions and Metabolism of Nutrients in Broiler. Master’s Thesis.

[8] Liu R., Jin C., Wang Z., Wang Z., Wang J., Wang L. (2015). Effects of manganese deficiency on the microstructure of proximal tibia and opg/rankl gene expression in chicks. Vet. Res. Commun..

[9] Liu X., Li Z., Han C., Zhang Z., Xu S. (2012). Effects of dietary manganese on Cu, Fe, Zn, Ca, Se, Il-1β, and Il-2 changes of immune organs in cocks. Biol. Trace Elem. Res..

[10] Wang Y.B., Chen F., Jiang S.Q., Gou Z.Y., Li L., Lin X.J., Fan Q.L., Cui X.Y. (2019). Dietary optimal supplemental level of manganese for Chinese yellow-feathered breeder hens during peak period of laying. Chin. J. Anim. Nutr..

[11] Ministry of Agriculture of the People’s Republic of China (2004). Chinese Chicken Feeding Standard.

[12] Li S., Luo X., Liu B., Crenshaw T.D., Kuang X., Shao G., Yu S. (2004). Use of chemical characteristics to predict the relative bioavailability of supplemental organic manganese sources for broilers. J. Anim. Sci..

[13] Wang Y.B., Li L., Gou Z.Y., Chen F., Fan Q.L., Lin X.J., Ye J.L., Zhang C., Jiang S.Q. (2020). Effects of maternal and dietary vitamin A on growth performance, meat quality, antioxidant status, and immune function of offspring broilers. Poult. Sci..

[14] Standardization Administration of China (2016). Method for Determination of Ash in Foods (GB 5009.4-2016).

[15] Eisemann J., Lewis H.E., Broome A.I., Sullivan K., Boyd R.D., Odle J., Harrell R.J. (2014). Lysine requirement of 1.5–5.5 kg pigs fed liquid diets. Anim. Prod. Sci..

[16] Pan S., Zhang K., Ding X., Wang J., Peng H., Zeng Q. (2018). Effect of high dietary manganese on the immune responses of broilers following oral *salmonella* typhimurium inoculation. Biol. Trace. Elem. Res..

[17] Jasek A., Coufal C.D., Parr T.M., Lee J.T. (2019). Evaluation of Increasing manganese hydroxychloride level on male broiler growth performance and tibia strength. J. Appl. Poult. Res..

[18] Gao Y.L. (2004). Studies on Manganese and Zinc Requirement of Gushi Chicks. Master’s Thesis.

[19] Ghosh A., Mandal G.P., Roy A., Patra A.K. (2016). Effects of supplementation of manganese with or without phytase on growth performance, carcass traits, muscle and tibia composition, and immunity in broiler chickens. Livest. Sci..

[20] Berta E., Andrásofszky E., Bersényi A., Glávits R., Gáspárdy A., Fekete S.G. (2004). Effect of inorganic and organic manganese supplementation on the performance and tissue manganese content of broiler chicks. Acta Vet. Hung..

[21] Alvaro M.B.J., Nelson L.M.F., Alessandra S., Alba F., Daiane H., Jovanir I.M.F. (2018). Arginine and manganese supplementation on the immune competence of broilers immune stimulated with vaccine against *Salmonella* Enteritidis. Poult. Sci..

[22] Luo X.G., Li S.F., Lu L., Liu B., Kuang X., Shao G.Z. (2007). Gene expression of manganese-containing superoxide dismutase as a biomarker of manganese bioavailability for manganese sources in broilers. Poult. Sci..

[23] Dodd C.A., Filipov N.M. (2011). Manganese potentiates LPS-induced heme-oxygenase 1 in microglia but not dopaminergic cells: Role in controlling microglial hydrogen peroxide and inflammatory cytokine output. Neurotoxicology.

[24] Hahon N., Booth J.A. (1984). Effect of chromium and manganese particles on the interferon system. J. Interferon Res..

[25] Ji F., Luo X.G., Lu L., Liu B., Yu S.X. (2006). Effects of manganese source and calcium on manganese uptake by in vitro everted gut sacs of broilers’ intestinal segments. Poult. Sci..

[26] Conly A.K., Poureslami R., Koutsos E.A., Batal A.B., Jung B., Beckstead R. (2012). Tolerance and efficacy of tribasic manganese chloride in growing broiler chickens. Poult. Sci..

[27] Liao X.D., Wang G., Lu L., Zhang L.Y., Lan Y.X., Li S.F., Luo X.G. (2019). Effect of manganese source on manganese absorption and expression of related transporters in the small intestine of broilers. Poult. Sci..

[28] Yuan Y., Hu Z.H. (2004). Effect of dietary manganese on the deposition of manganese, copper and zinc in tissues and organs of broilers. Anim. Husb. Vet. Med..

[29] Liu C.H., Heinrichs B.S., Leach R.M. (1994). Influence of manganese deficiency on the characteristics of proteoglycans of avian epiphyseal growth plate cartilage. Poult. Sci..

[30] Noetzold T.L., Vieira S.L., Favero A., Horn R., Silva C.M., Martins G.B. (2020). Manganese requirements of broiler breeder hens. Poult. Sci..

[31] Oliveira T.F.B., Bertechini A.G., Bricka R.M., Kim E.J., Peebles E.D. (2015). Effects of in ovo injection of organic zinc, manganese, and copper on the hatchability and bone parameters of broiler hatchlings. Poult. Sci..

[32] Lu L., Luo X.G., Ji C., Liu B., Yu S.X. (2006). Effect of manganese supplementation and source on carcass traits, meat quality, and lipid oxidation in broilers. J. Anim. Sci..

[33] Zhu Y., Lu L., Liao X., Li W., Luo X. (2017). Maternal dietary manganese protects chick embryos against maternal heat stress via epigenetic-activated antioxidant and anti-apoptotic abilities. Oncotarget.

[34] Zhang X., Wang B., Ge W., Zhang M., Yue B., Shi X., Wang X., Xu C. (2014). Effects of manganese on serum biochemical indexes, tissue manganese deposition, antioxidant ability and tibia development of Wulong geese aged from 5 to 16 weeks. Chin. J. Anim. Nutr..

